# Establishment of a glucocorticoid inducible system for regulating somatic embryogenesis in *Liriodendron* hybrids

**DOI:** 10.48130/forres-0024-0003

**Published:** 2024-02-29

**Authors:** Xinying Chen, Ye Liu, Lu Lu, Siqin Liu, Yuhao Weng, Jisen Shi, Zhaodong Hao, Jinhui Chen

**Affiliations:** 1 State Key Laboratory of Tree Genetics and Breeding, Co-Innovation Center for Sustainable Forestry in Southern China, Nanjing Forestry University, Nanjing 210037, China; 2 Key Laboratory of Forest Genetics and Biotechnology of Ministry of Education, Nanjing Forestry University, Nanjing 210037, China

**Keywords:** Glucocorticoid inducible expression system, *Liriodendron* hybrids, *LhWUS*

## Abstract

The precise expression of transcription factors (TFs) is crucial for plant growth and development, especially during somatic embryogenesis. However, conventional overexpression approaches, commonly used for functional genetics, can lead to deleterious effects. Therefore, it is imperative to ensure that TFs are expressed in a controlled and timely manner when aiming to enhance the efficiency of somatic embryogenesis. In this study, a dexamethasone/glucocorticoid receptor (DEX/GR) inducible expression system was employed to modulate the protein expression levels of target TFs within the nucleus during somatic embryogenesis in *Liriodendron* hybrids. We selected the *WUSCHEL* (*WUS*) gene, a well-established functional TF known for its vital role in somatic embryogenesis, as a model to assess the effectiveness of this system. Through DEX treatment, we induced the translocation of LhWUS-GR/LhWUS-GFP-GR fusion proteins from the cytoplasm to the nucleus, consequently leading to *WUS*-driven somatic embryogenesis. As the DEX concentration increased, there was a corresponding increase in the migration of the LhWUS-GFP-GR fusion protein into the nucleus. Additionally, we observed a higher proliferation ratio of callus expressing *LhWUS-GR* when exposed to varying DEX concentrations. Notably, the efficiency of somatic embryogenesis exhibited significant improvement under optimal DEX concentration. In conclusion, our study successfully utilizes the DEX/GR inducible system in *Liriodendron* hybrids, offering a valuable tool for the precise control and utilization of TFs at the desired levels. This innovative approach holds promise for advancing our understanding of TF function and enhancing plant development through the regulated manipulation of TF expression.

## Introduction

Since the introduction of the first transgenic crop in 1983, rapid advancements have been made in the field of transgenic technology, and the generation of transgenic plants has become a regular practice. The 35S promoter, derived from the cauliflower mosaic virus, is a widely used constitutive promoter applied in transgenics to overexpress a desired gene^[1]^. However, the 35S promoter possesses certain limitations. It cannot be employed for genes that induce lethality, and it cannot target expression to specific developmental stages or tissues. To overcome these limitations, research efforts have focused on developing inducible promoters. Numerous chemically regulated promoter systems have been developed for in planta expression^[2,3]^. The most widely used systems include tetracycline-dependent expression^[4,5]^, glucocorticoid-inducible expression^[6]^, dual control induction^[7]^, and ethanol-inducible expression^[8]^.

Glucocorticoid-inducible expression systems consist of two key components: a transcription factor (TF) and a response element^[9]^. The aforementioned TF glucocorticoid receptor (GR) is a vertebrate steroid hormone receptor. The GR-dexamethasone (DEX) system has proven to be a reliable plant induction system, as DEX acts as a potent synthetic glucocorticoid that does not elicit any pleiotropic effects in plants^[2,6,10]^. Under DEX-free conditions, TF-GR forms a cytoplasmic complex with HSP90, a heat shock protein. The binding of DEX to GR triggers the dissociation of HSP90, leading to the nuclear localization of TF-GR. Once in the nucleus, GR is capable of activating the transcription of glucocorticoid response elements (GREs) that are cloned into the promoter region of the expression vector used for transformation, thus controlling transcription of the target gene both spatially and temporally^[11,12]^.

It is crucial to ensure that components of an inducible expression system have no significant off-target effects on the organism's biology. In the glucocorticoid-inducible expression system, both the TF and DEX are foreign to plants, making them ideal components for such a system. The GR system is the most widely used, and it has been applied to a variety of species such as *Arabidopsis thaliana*^[13,14]^, *Nicotiana tabacum*^[9,15]^, *Oryza sativa*^[16]^, *Malus × domestica*^[17]^, *Lotus corniculatus*^[18]^ and *Lactuca sativ*a^[10]^. However, several studies have shown that this system is associated with some shortcomings. Given that DEX possesses a certain degree of toxicity, the plant's growth is impeded after treatment, indicating DEX alone can induce expression of defense-related genes^[19,20]^.

*Liriodendron* contains two sister species: *Liriodendron chinense* and *Liriodendron tulipifera*^[21]^. *Liriodendron* hybrid is the product of interspecific hybridization between *L. chinense* and *L. tulipifera*, and it displays remarkable hybrid vigor^[22]^. Valued for its ornamental and economical significance, the *Liriodendron* hybrid exhibits favorable traits such as excellent timber quality, resilience to stress, wide adaptability, rapid growth, beautiful flowers, vibrant colors, and appealing tree shape^[23]^. Currently, extensive research efforts are being made on the molecular breeding of various *Liriodendron* hybrid combinations^[24,25]^. Plant somatic embryogenesis, characterized by its rapid growth rate and high reproductive efficiency, serves as an effective approach for plant regeneration and rapid propagation^[26,27]^. The birth of the somatic embryogenesis method in *Liriodendron* provides an efficient way to popularize these high-value trees. Simply relying on persistent overexpression is not conducive to studying gene function in forestry due to its complex genetic background. A stable and precise inducible expression system is necessary for the study of gene function, strengthening gene function, and artificially regulating the expression of key genes in a timed and quantitative manner.

Therefore, to successfully obtain transgenic plants and accurately regulate the expression of foreign genes, we aimed to establish a chemical DEX/GR induction system in *Liriodendron*. We selected the *Liriodendron* hybrid *WUSCHEL* (*LhWUS*) gene as a test molecule to study the efficacy of our GR expression vector system. In plants, TFs play an irreplaceable role in the regulation of regeneration and somatic embryogenesis^[28,29]^. While TFs are required for these developmental processes, their overexpression can have deleterious effects on plant growth^[30]^. *WUSCHEL* has been previously reported to regulate the development of somatic embryos in *Arabidopsis*^[31]^. However, *WUS* overexpression in *Picea glauca* hampers seedling growth^[32]^. Similarly, in cotton (*Gossypium hirsutum*) callus, *WUS* overexpression can stimulate the formation of somatic embryos, albeit the resulting calli are not regenerative^[33]^. By adding different concentrations of specific chemical inducers, we aimed to precisely control the expression level of exogenously expressed *LhWUS*. Through assessment of the expression level, protein localization, and *LhWUS* overexpression phenotype, we demonstrate that the DEX/GR system is highly responsive in *Liriodendron,* and provide a valuable tool for further molecular work in this species*.*

## Materials and methods

### Plant materials and growth conditions

Embryonic calli were induced from immature zygotic embryos of the *Liriodendron* hybrid (*L. chinense* × *L. tulipifera*)^[34]^. The calli were subcultured on ¾ MS medium containing 2 mg·L^−1^ 2,4-Dichlorophenoxyacetic acid (2,4-D) in a dark environment at 23 °C. The succession cycle was set as 25 d. To induce somatic embryos, calli were placed on ¾ MS medium without 2,4-D. After 45 d of induction, mature somatic embryos with cotyledons were observed and counted. These somatic embryos were exposed to light for 7 d and transferred to ¾ MS medium containing 2 mg·L^−1^ Indole butyric acid (IBA) for 2 months to obtain *Liriodendron* hybrid regenerated plants.

### Plasmid construction and genetic transformation

We identified *LhWUS* based on the genomic data of *Liriodendron*^[23]^ through homology with *Arabidopsis thaliana WUS* (www.ncbi.nlm.nih.gov/). The identified *LhWUS* sequence was used as the reference to design primers for cloning. We generated cDNA from RNA extracted from the *Liriodendron* hybrid shoots and used the reverse complement as the template for cloning *LhWUS*. We cloned *LhWUS* using Phanta (Vazyme Biotech, China) and constructed vectors *via* ClonExpress II (Vazyme Biotech). The *GR* sequence was cloned and constructed into the pBI121 basic vector using a recombination technique, and it served as a control in this study. The *pBI121:LhWUS-GR* vector was constructed by inserting the *LhWUS* gene into the *pBI121:GR* vector using *Bam*HI (NEB, USA). To construct the *pBI121:LhWUS-GFP-GR* vector, we cut the vector *PBI121:LhWUS-GR* with *Xma*I (NEB) and concatenated the *GFP* gene into the vector *via* recombinant ligation. The primer sequences are listed in Supplemental Table S1. *Escherichia coli* DH5α strain (Tiangen, China) and *Agrobacterium tumefaciens* EHA105 strain (Weidi, China) were used for vector construction and transformation, respectively. Light yellow embryogenic calli were selected for transformation, and transgenic calli were obtained through G418 antibiotic screening at a concentration of 90 mg·L^−1^^[24,35]^. Positive transgenic calli were harvested after several subcultures, and vector expression was determined by quantitative polymerase chain reaction (qPCR).

### Expression analyses and qPCR

Approximately 0.1 g of *Liriodendron* hybrid transgenic callus was utilized for total RNA extraction using the Eastep Super Total RNA purification kit (Promega, USA), according to the manufacturer's instructions. cDNA was generated using the HiScript III First Strand cDNA synthesis kit (+gDNA wiper) (Vazyme Biotech). The amplification reactions were conducted on a LightCycler 480 qPCR detection system (Roche, Switzerland) using TB Green Premix Ex Taq (Takara, China), according to the manufacturer's instructions. The expression of the *LhWUS* gene was normalized to that of the *LhUBQ* gene, and the target gene expression levels were quantified using the 2^−ΔΔCᴛ^ method^[36]^. The primers are provided in Supplemental Table S1.

### Dexamethasone treatments

We stored DEX in absolute ethanol at −20 °C at a concentration of 10 mM. To apply DEX to plants, an appropriate amount of DEX stock was diluted in an appropriate amount of medium.

### Confocal microscopy

To investigate the response time of the DEX/GR system, we viewed the treatment process under confocal microscopy. Calli were resuspended in PBS at an appropriate dilution. Next, 5 μL of the suspension was placed on a glass slide for imaging. The GFP signal was observed at an excitation wavelength of 488 nm (pinhole: 1.00 AU/30 μm; laser wavelength: 3.90%; gain: 732 V). We used the Z-stack mode to obtain three-dimensional fluorescence images of gene expression and protein localization. We scanned hierarchical fluorescence images at 1 min intervals following DEX treatment and captured continuous photographs of the same cells. This approach allowed us to observe cells undergoing treatment by the DEX/GR system.

The cells of the transgenic callus were stained using Hoechst 33342 (Sangon Biotech, China) following DEX treatment. In brief, the cells were fixed with 3.7% formaldehyde, followed by centrifugation for 5 min. The supernatant was removed, and the cells were washed twice with PBS. The cells were then treated with 0.2% Triton X-100, and the supernatant was removed after 2 min. Hoechst 33342 was added for staining, and the cells were stained for 1–2 min. After staining, the cells were washed twice with an appropriate amount of PBS. The nuclear localization signal was observed at an excitation wavelength of 405 nm (pinhole: 1 AU/30 μm; laser wavelength: 4.5%; gain: 700 V).

### Fluorescence intensity quantification

The fluorescence intensity was measured using the irregular measurement tool within Zeiss Zen software (Carl Zeiss, Germany), which allowed us to select regions of interest displaying GFP fluorescence within the nucleus. We collected average gray values for the GFP fluorescence of the same locations before and after treating the cells with DEX to assess gene expression in the nucleus.

### Statistical analysis

After capturing images of the shoot apical meristem of both treated and untreated *LhWUS-GR* plants, the shoot apical meristem area was calculated using ImageJ software (National Institutes of Health, USA)^[37]^. We used Student's *t*-test with *p*-value cutoffs of <0.05 to determine statistical significance.

## Results

### LhWUS-GFP-GR fusion protein enters the nucleus in DEX-treated *Liriodendron* hybrid callus

**Figure 1:**
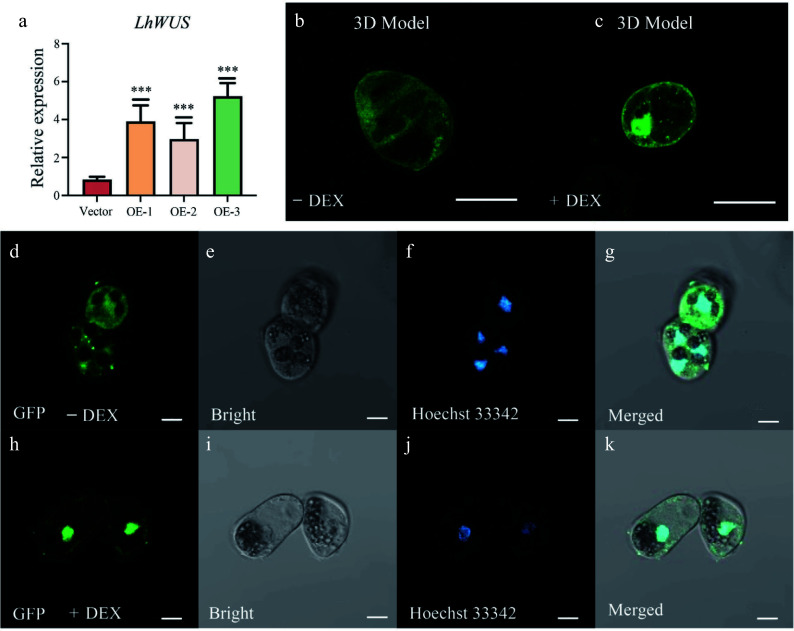
Overexpression of *p35S:LhWUS-GFP-GR* gene in *Liriodendron* hybrid callus cells. (a) *LhWUS-GFP-GR* transgenic calli and empty qPCR was used to assess the expression of the *LhWUS* gene. (b), (d)–(g) *p35S*:*LhWUS-GFP-GR* transgenic *Liriodendron* hybrid callus cells without DEX treatment. (c), (h)–(k) *p35S:LhWUS-GFP-GR* *Liriodendron* hybrid callus cells treated with 10 μM DEX for 7 d. The results were generated under the following conditions: (b), (d), (h) under a wavelength of 488 nm, (e), (i) under bright field, (f), (j) under a wavelength of 405 nm, (g), (k) the merging of three images, and (b), (c) Z-stacks were used to shoot and set the image acquisition range. Fluorescence scanning was performed at every set value to obtain a three-dimensional fluorescence image. The final presentation consisted of a superimposed three-dimensional image. The expression was measured using *LhUBQ* as the internal reference gene. Scale bar: (b), (c) 40 μm; (d)–(k) 10 μm. ***, *p* < 0.01. Data represent mean ± SD from three biological replicates.

To explore whether the DEX/GR based transgenic induction system would be functional in *Liriodendron*, we constructed a *p35S:LhWUS-GFP-GR* vector and introduced it into *Liriodendron* hybrid embryogenic callus using a previously reported method^[24]^. Transgenic calli were harvested using antibiotic G418 screening and further verified through qPCR. Recovered calli possessed a light-yellow color and fine texture, revealing their suitability for further research. Transgenic calli were collected from three different lines overexpressing *LhWUS-GFP-GR* for qPCR. Our findings revealed that the transgenic lines exhibited a 3–6 fold increase in the expression of *LhWUS* at the callus stage compared to the control (Fig. 1a). To induce the entry of the LhWUS-GFP-GR fusion protein into the nucleus, we transferred the *p35S:LhWUS-GFP-GR* transgenic callus to media containing 10 μM DEX, using untreated callus (without DEX, mock) as the control. Three-dimensional scanning provided a more intuitive image of the green fluorescence in the nucleus after DEX treatment (Fig. 1b, c). After fixing with formaldehyde, we stained the treated and untreated calli with Hoechst 33342. The stained cells exhibited blue fluorescence at a wavelength of 405 nm. Subsequently, we viewed the calli by confocal microscopy and observed that GFP fluorescence was uniformly distributed in the cytoplasm in the control (Fig. 1d–g), while the GFP signal converged in the nucleus in DEX-treated calli (Fig. 1h–k). These results suggest that the GR system functions effectively in *Liriodendron* hybrid callus following DEX-mediated induction.

**Figure 2:**
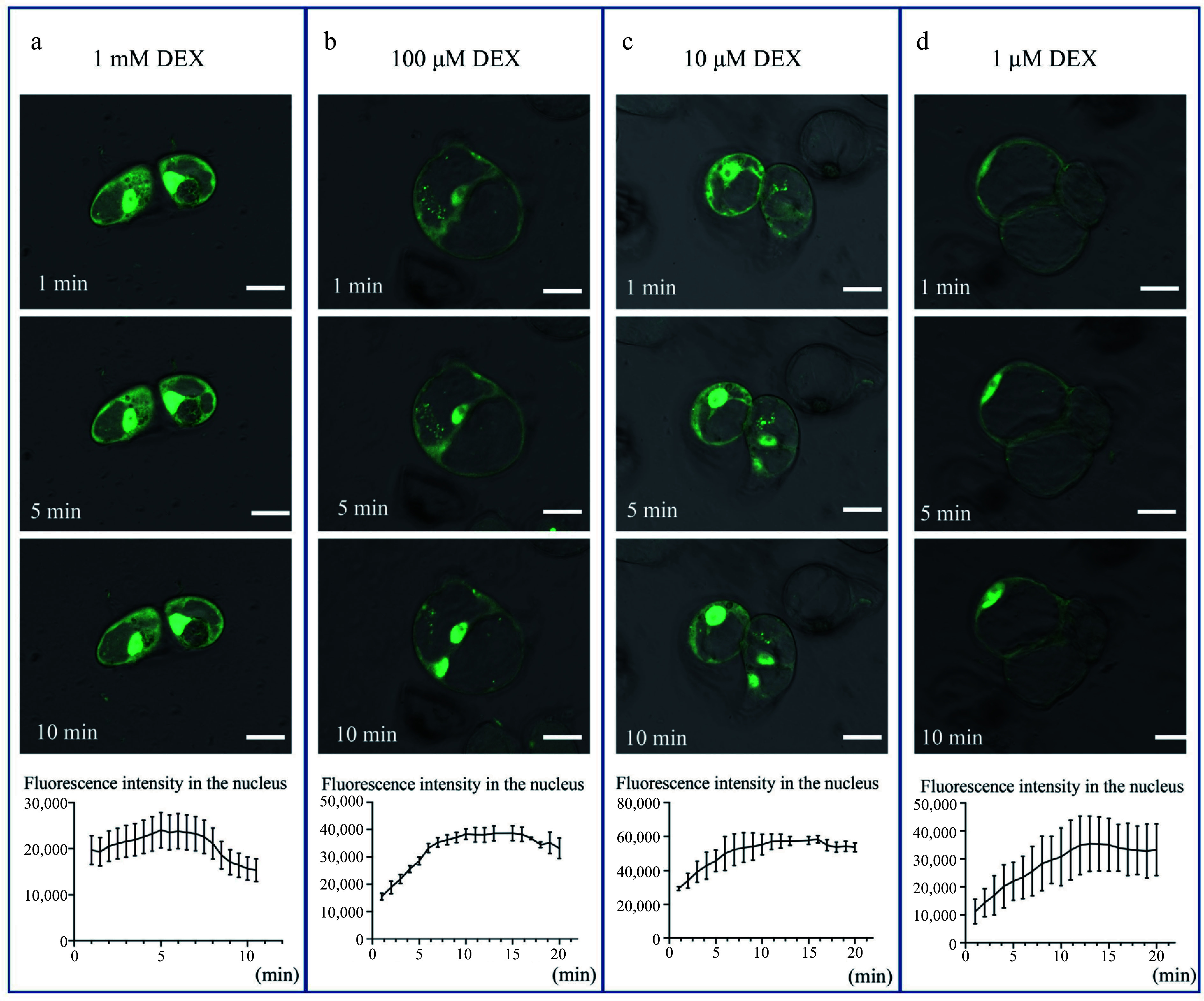
Dynamic 3D images of *p35S:LhWUS-GFP-GR* transgenic *Liriodendron* hybrids callus under confocal laser. After treating the *LhWUS-GFP-GR* transgenic cells with different concentrations of DEX, sequential images of the same cells were obtained using Z-stacks. Images were acquired every minute, with each set of images arranged from top to bottom, representing photos taken at 1 min, 5 min, and 10 min after the cells were treated. ZEN software was used to outline the nucleus, followed by quantitative analysis of the fluorescence intensity. The results of treating transgenic cells with (a) 1 mM DEX, (b) 100 μM DEX, (c) 10 μM DEX, and (d) 1 μM DEX were obtained. Scale bar = 20 μm.

To further investigate the translocation of the LhWUS-GFP-GR fusion protein into the nucleus after DEX treatment, we used increasing DEX concentrations (1 μM, 100 μM, 10 μM, and 1 mM) and examined whether increased DEX application would result in more rapid translocation. In brief, *p35S:LhWUS-GFP-GR* transgenic callus was immersed in ¾ MS medium supplemented with DEX, and the fluorescence signal in the nucleus was measured every minute. When treated with 1 mM DEX, the GFP fluorescence signal in the nucleus peaked within 5 min and then rapidly declined (Fig. 2a). Conversely, with the three lower concentrations, a 10–15 min lag period was observed prior to peak fluorescence intensity in the nucleus, after which the signal gradually declined (Fig. 2b–e). These findings illustrate the rapid response of the GR system in *Liriodendron* hybrid cells. Nonetheless, it is crucial to determine the optimal concentration to ensure sustained effectiveness when utilizing a chemical induction system.

### DEX-treated *WUS-GR* *Liriodendron* hybrid callus promotes callus proliferation

**Figure 3:**
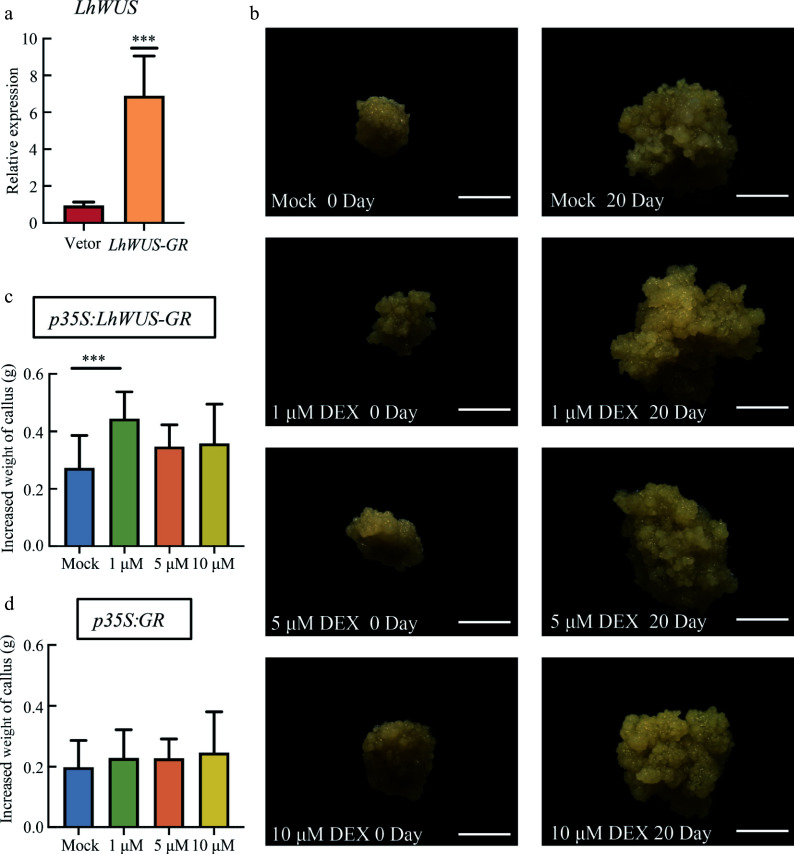
Transgenic callus treated with different concentrations of DEX. (a) qPCR was used to detect the expression of the *LhWUS* gene in *LhWUS-GR* transgenic calli, with the empty vector serving as the control. (b) The phenotype of *LhWUS-GR* transgenic callus was observed before and after treatment with different concentrations of DEX. The left column represents the phenotype before treatment, whereas the right column represents the phenotype after treatment. Scale bar = 5 mm. (c), (d) Transgenic callus treated with different concentrations of DEX for 20 d resulted in an increase of the callus weight. ***, *p* < 0.01. Data represent mean ± SD from three biological replicates.

To ensure that GFP fusion proteins do not interfere in induction, we introduced *p35S:LhWUS-GR* and *p35S:GR* vectors into *Liriodendron* hybrids. Prior to processing the calli, we collected calli for qPCR. The expression of *LhWUS* in the transgenic calli overexpressing *LhWUS-GR* increased by 4–8 times compared to the control, confirming the transgenic nature of the plants (Fig. 3a). To investigate the impact of DEX on callus growth, we conducted a comparative study using different concentrations of DEX (mock, 1 μM, 5 μM, and 10 μM) on *p35S:LhWUS-GR* and *p35S:GR* transgenic callus (Fig. 3b−d). After a 25 d treatment period, the calli were weighed. Statistical analysis revealed that DEX treatment at varying concentrations did not have a significant effect on *p35S:GR* callus weight, and we observed no significant difference between the treatment groups. However, compared to the control, the weight of *p35S:LhWUS-GR* transgenic calli increased after DEX treatment, with a significant difference observed at a concentration of 1 μM. Callus weight also increased after treatment with 5 μM and 10 μM DEX compared to the control, but the results were not statistically significant. These findings suggest that an appropriate amount of DEX can enhance proliferation and growth of *p35S:LhWUS-GR* transgenic callus.

### Embryo regeneration from *LhWUS-GR* transgenic callus

**Figure 4:**
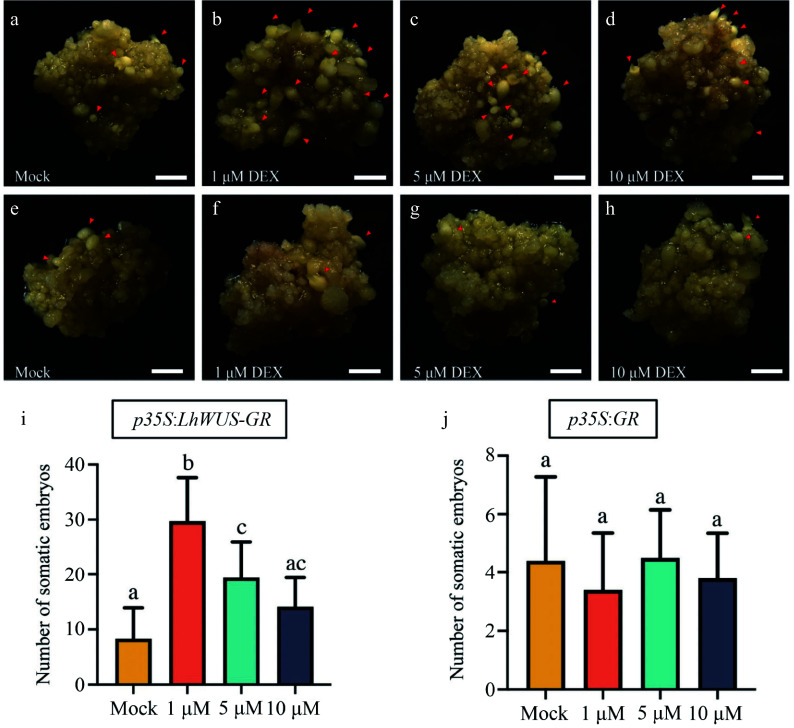
Phenotypes and statistics of the number of somatic embryos induced on medium containing ABA after treatment with different concentrations of DEX. (a), (e) Transgenic calli were induced using the same amount of absolute ethanol as in the control group. (b), (f) Transgenic calli induced the somatic embryo phenotype after treatment with 1 μM DEX. (c), (g) Transgenic calli showed the phenotype of somatic embryos induced by 5 μM DEX. (d), (h) Transgenic calli showed the phenotype of somatic embryos induced by 10 μM DEX. (i), (j) The average number of somatic embryos per callus exposed to different concentrations of DEX; Scale bar = 1 mm. The letters a–d denote statistical significance (*one-way ANOVA, Tukey's multiple comparisons test*.

To further study the role of the DEX/GR system in somatic embryogenesis, we transferred the same weight of DEX-treated *p35S:LhWUS-GR* and *p35S:GR* callus tissue to ¾ MS solid medium for induction of somatic embryogenesis. This was done to induce somatic embryogenesis after treating the tissue with various concentrations of DEX (Fig. 4a−h). After 45 d of induction culture, we observed the formation of somatic embryos in both the treatment and control groups. Statistical analysis revealed that the number of somatic embryos produced by DEX-treated *p35S:LhWUS-GR* transgenic calli was higher than that in the untreated group (Fig. 4i). Specifically, *p35S:LhWUS-GR* transgenic calli treated with DEX at a concentration of 1 μM were able to produce more somatic embryos compared to the control. The *p35S:LhWUS-GR* transgenic calli treated with DEX at a concentration of 5 μM also showed significant differences compared to the control, although the number of somatic embryos in this group was significantly lower than that in the group treated with 1 μM. There was no significant difference in the somatic embryogenesis of *p35S:GR* callus after treatment with various concentrations of DEX (Fig. 4j). Therefore, our findings suggest that the DEX/GR system may play a dose-effect role in regulating the LhWUS during somatic embryogenesis.

### Phenotypes produced by *LhWUS-GR* plants following DEX treatment

**Figure 5:**
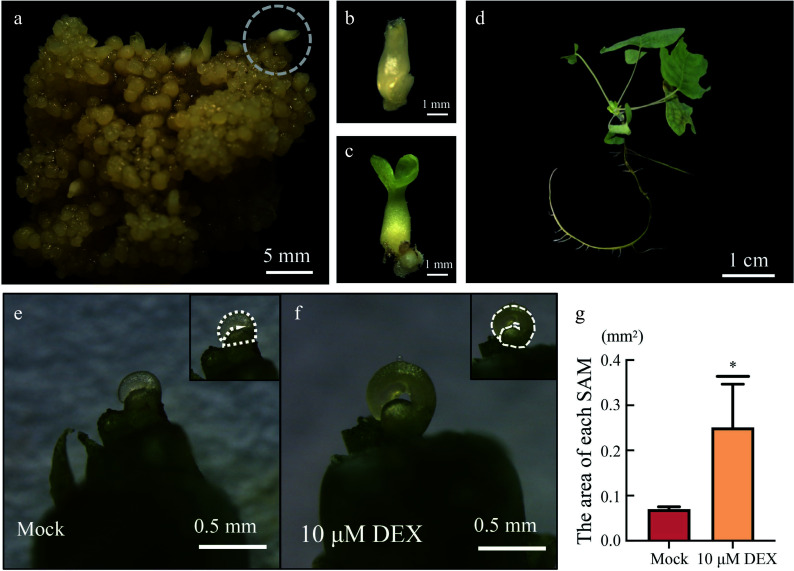
Induction of transgenic plants by solid culture. (a) *LhWUS-GR* callus was solid-induced for 45 d on ¾ MS medium. (b) Cotyledon embryos detached from the callus. (c) Cotyledon embryos after 7 d of light. (d) Two-month-old plantlets grown from regenerated cotyledon embryos. (e) Meristem of *LhWUS-GR* transgenic plants treated with mock for 10 d. (f) *LhWUS-GR* transgenic plants of shoot tip treated with 10 μM DEX for 10 d. (g) Statistics on the area size of SAM of *LhWUS-GR* plants treated with mock and 10 μM DEX. Scale bar: (a) 5 mm; (b), (c) 1 mm; (d) 1 cm; (e), (f) 0.5 mm. *, *p* < 0.05. Data represent mean ± SD from three biological replicates.

*p35S:LhWUS-GR* transgenic callus was cultured in a light incubator after solid culture. After one week of light exposure, the somatic embryos further matured, resembling cotyledon embryos, but without the radicle. The somatic embryos were detached from the cotyledon embryo stage of transgenic calli (Fig. 5a−c) and transferred to rooting medium containing IBA for further culture. After one month, the majority of regenerated plants derived from the somatic embryos rooted and continued to grow into adult plants (Fig. 5d), indicating that somatic embryos formed by DEX-induced *p35S:LhWUS-GR* transgenic callus could successfully develop into full plants.

To investigate the effectiveness of the DEX/GR system in transgenic plants, we cultivated *p35S:LhWUS-GR* somatically regenerated plants on ¾ MS solid medium for continued growth. Ten *LhWUS-GR* plants, each with 3–4 leaves, were then transferred to ¾ MS solid medium supplemented with 10 μM DEX for chemical induction. As a control, *p35S:LhWUS-GR* plants of similar size were placed on ¾ MS solid medium containing the same concentration of ethanol. After 10 d of induction treatment, we removed the congenital stipules of the *Liriodendron* hybrid and observed the apical meristems. Our findings revealed that the apical meristems of *p35S:LhWUS-GR* transgenic plants exhibited a noticeable enlargement after DEX chemical induction compared to the apical meristems of untreated *p35S:LhWUS-GR* transgenic plants (Fig. 5e–g). This observation was consistently observed in three independent experiments, indicating that DEX/GR can effectively influence the growth of *Liriodendron* hybrid plants.

## Discussion

Here, we establish a chemically inducible gene expression system in *Liriodendron,* generating a critical tool for genetic resolution in this economically relevant tree species*.* Our DEX/GR system effectively regulated the localization of our test TF-GR fusion protein within the nucleus. However, DEX/GR inducible systems face some limitations. Notably, DEX is a toxic reagent that can cause growth retardation and obstruct developmental phenotyping. Additionally, DEX induces expression of defense-related genes, potentially interfering with disease phenotyping^[19]^. It is necessary to determine whether the observed phenotypes are the result of DEX treatment or the expression of target genes. In this study, the efficiency of somatic embryogenesis in *35S:LhWUS-GR* calli, even without DEX treatment, was slightly higher than that of *35S:GR* calli. We hypothesize that this difference may be attributed to leaky expression of TF, a common phenomenon in chemical systems^[38]^.

Chemical induction systems are widely used to study plant regeneration, especially to elucidate the function of genes involved in somatic embryogenesis. The chimeric transcription activator, XVE, was created by combining the DNA-binding domain of the bacterial repressor LexA (X), the acidic transactivating domain of VP16 (V), and the regulatory region of the human estrogen receptor (E; ER)^[39]^. In *Arabidopsis thaliana*, *AtWUS* expressed under the XVE system was found to promote the somatic to the embryogenic transition, allowing for plant regeneration after removal of the inducer^[40]^. In tobacco^[41]^ and sweet pepper (*Capsicum frutescens*)^[42]^, *BABY BOOM-GR* enhanced the regeneration ability by chemical induction systems.

Furthermore, chemical induction systems offer the potential for targeted transcriptomic, metabolomic, and proteomic analyses. The GR-tag has been successfully used in chromatin immunoprecipitation (ChIP) experiments^[43]^. Affinity-purified polyclonal anti-GR antibodies can be utilized for ChIP enrichment. Subsequently, validate the interaction of candidate target genes using ChIP-qPCR or ChIP-seq techniques. RNA sequencing or chromatin immunoprecipitation of plants expressing a target TF under the control of the DEX/GR system can reveal novel TF targets, establishing biological roles for these TFs^[44]^.

Given that *WUS* overexpression promotes cell proliferation^[45]^, we transferred *p35S:LhWUS-GR* into *Liriodendron* hybrid embryogenic callus and leveraged calli phenotyping to assess the efficacy of our system. We found that callus weight and LhWUS-GR fusion protein translocation into the nucleus were increased with higher DEX concentrations. The results of this study demonstrate that the DEX/GR chemical induction system can be effectively employed in *Liriodendron* hybrid callus. More importantly, this inducible system allows for expression of exogenous genes that may have lethal effects when constitutively expressed. This system lays a foundation for functional genetics in *Liriodendron* hybrids and provides a model that can be applied to other species.

## Conclusions

In this study, we describe an inducible DEX/GR gene expression system that can control somatic embryogenesis in *Liriodendron* hybrids. *WUSCHEL,* an endogenous TF responsible for regulating somatic embryogenesis, was expressed under the control of the DEX/GR system, and achieved conditional translocation into the nuclei of calli*.* *WUS* expression produced somatic embryos in a dose-dependent manner with DEX treatment. The most effective concentration for both callus proliferation and subsequent somatic embryo induction was found to be 1 μM. Furthermore, this system retained its functionality in regenerated plants. In summary, this work establishes the DEX/GR system as a feasible and an efficient method for the functional exploration and utilization of TFs in *Liriodendron*, and possibly related species.

## SUPPLEMENTARY DATA

Supplementary data to this article can be found online.

## Data Availability

The *LhWUS* (accession number: Lchi22683) sequence can be downloaded from the *Liriodendron* *chinense* genome (https://db.cngb.org/search/assembly/CNA0007303/). All data generated or analyzed during this study are included in this article and its supplementary files.
